# Seroprevalence of *Rhodococcus equi* in horses in Israel

**DOI:** 10.4102/jsava.v88i0.1508

**Published:** 2017-06-26

**Authors:** Sharon Tirosh-Levy, Sevil E. Gürbilek, Osman Y. Tel, Oktay Keskin, Amir Steinman

**Affiliations:** 1 Koret School of Veterinary Medicine, The Hebrew University of Jerusalem, Israel; 2 Department of Microbiology, Harran University, Eyyubiye Campus, Turkey

## Abstract

*Rhodococcus equi* is a common cause of pneumonia in foals and has extensive clinical, economic and possibly zoonotic consequences. This bacterium survives well in the environment and may be considered as normal flora of adult horses. Certain strains of this bacterium are extremely virulent in foals, and early identification and intervention is crucial for prognosis. *Rhodococcus equi* is endemic in many parts of the world and occasionally isolated in Israel. This study was designed to evaluate *R. equi* seroprevalence in adult horses in Israel to indirectly indicate the potential level of exposure of susceptible foals. Sera were collected from 144 horses during spring 2011 and from 293 horses during fall 2014, and the presence of antibodies against virulent *R. equi* was detected by enzyme-linked immunosorbent assay. Equine seroprevalence of *R. equi* was found to be 7.6% in 2011 and 5.1% in 2014. Only one farm had seropositive horses in 2011, whereas several farms had seropositive horses in 2014. No significant risk factors for seropositivity were found. *Rhodococcus equi* appears to be endemic in Israel. This is the first survey of *R. equi* in Israel that provides information on the epidemiology of this important bacterium.

## Introduction

*Rhodococcus equi* is a common cause of pneumonia in foals and has extensive clinical and economic consequences. Clinical disease typically affects foals between 3 weeks and 6 months of age on endemic farms (Reuss & Cohen 2015). The main clinical manifestation is chronic suppurative bronchopneumonia with lung abscesses, although many cases are accompanied by various extra-pulmonary disorders, including ulcerative enterocolitis, joint effusion and ocular disorders (Giguère et al. 2011a). Morbidity and mortality in foals are high, whereas healthy adult horses are rarely diagnosed with clinical disease (Cohen 2014; Giguère et al. 2011a, 2011b; Morresey & Waldridge 2010; Muscatello 2012a; Vazquez-Boland et al. 2013). In recent years, the awareness of the zoonotic potential of *R. equi* has increased, mainly in immunocompromised human patients, thus emphasising the importance of better surveillance of this pathogen (Ocampo-Sosa et al. 2007; Vazquez-Boland et al. 2013; Weinstock & Brown 2002).

*Rhodococcus equi* is a Gram-positive facultative intracellular bacterium that is saprophytic in soil and multiplies in herbivore faeces. The main reservoir is in the soil, but it can also replicate in equine intestines (Giguère et al. 2011b; Muscatello 2012a). Transmission is faecal–oral, yet spread via aerosol plays an important role in clinical infection. Only certain strains of *R. equi* are pathogenic, and several virulence genes and plasmids have been identified. In the environment, most *R. equi* isolates lack virulence traits, but under selection in the host environment acquisition of virulent plasmids may occur, and more virulent isolates are found. The epidemiology of *R. equi* exhibits a delicate balance between the environment, subclinical carriers and susceptible hosts. The main reservoir is in soil, which makes it endemic in many equine facilities, but it also appears sporadically in non-endemic locations. The risk of infection increases with animal density and dusty hot dry climates (Muscatello 2012a; Vazquez-Boland et al. 2013).

Diagnosis and prevention are challenging, and because early detection of clinical cases improves survival, much effort has been put into developing effective surveillance programmes. The gold standard is isolation of bacteria from trans-tracheal lavage of a suspected case and the detection of the virulence-associated plasmid A (VapA) gene with the aid of polymerase chain reaction (PCR) (Muscatello 2012b; Reuss & Cohen 2015). But, because *R. equi* is mainly an environmental organism and possibly a commensal in healthy horses, isolation of bacteria does not always correlate with clinical disease and isolation of the bacterium must be accompanied with septic lung cytology or ultrasonographic imaging of lung abscessation (Cohen 2014; Giguère et al. 2011b; Reuss & Cohen 2015). Attempts at screening and early detection have been done with routine auscultations and ultrasonography, which, although widely used worldwide, are labour intensive and nonspecific (Cohen 2014; Giguère et al. 2011b; Muscatello 2012b). Serological screening methods, mainly enzyme-linked immunosorbent assay (ELISA), using whole bacterium, crude antigens and lately VapA specific antigens, were developed for early detection of exposure to *R. equi* (Giguère et al. 2011b; Reuss & Cohen 2015). In general, most serological tests for antibodies against *R. equi* have low specificity and cannot discriminate between subclinical infection and maternally derived antibodies (Giguère & Prescott 1997; Giguère et al. 2011b; Higuchi et al. 1997). Several studies in recent years attempted to evaluate and improve the sensitivity and specificity of ELISA screening, and to determine the cut-off values for early detection of clinical cases (Giguère et al. 2003; Martens et al. 2002; Sanz et al. 2014; Witkowski et al. 2012). Despite these limitations, serological surveys may be of value in identifying endemic farms and assessing the potential for clinical infection in susceptible animals (Attili et al. 2006; Sanada, Noda & Nagahata 1992; Tel, Arserim & Keskin 2011). A serological survey of subclinically infected horses in Japan demonstrated that *R. equi* seroprevalence in farms may differ between clinically endemic, sporadic and ‘clean’ farms (Sanada et al. 1992).

In Israel, *R. equi* was first reported in two 2-month-old foals in 1998 (Steinman, Sutton & Elad 1999). Since then, the bacterium had been isolated from trans-tracheal lavage fluids in a few foals suffering from bronchopneumonia (one in 2011, none in 2012, one in 2013 and none in 2014 and 2015 http://www.vetserv.moag.gov.il/Vet/all_Publications/dochot-shnatiim/default.htm, accessed April 2017). Although climatic conditions and horse management in Israel are suitable for this bacterium, little is known about its prevalence. This study was conducted to assess the prevalence of *R. equi* in adult horses in Israel by using crude protein antigen–based ELISA, in order to indirectly indicate the level of exposure of foals, as access to foals for sampling was very limited.

## Materials and methods

### Sample collection

**FIGURE 1:**
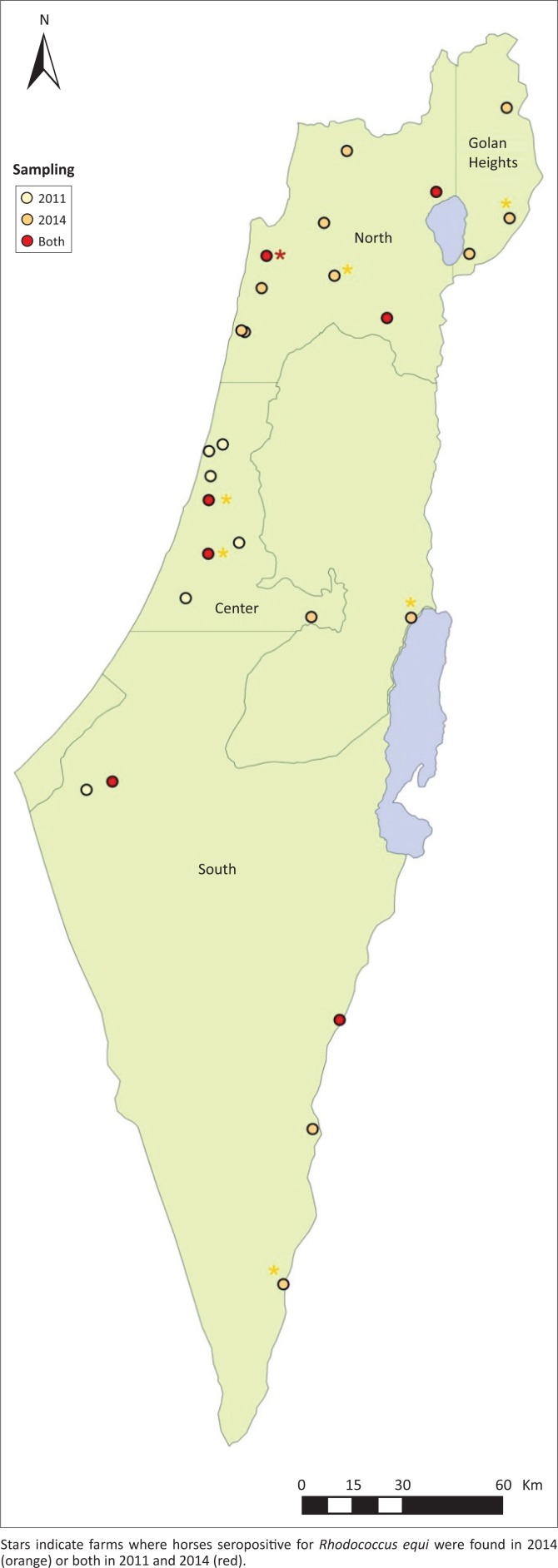
Geographic distribution of the farms sampled during 2011 (yellow), 2014 (orange) or both (red).

Serum samples were collected from apparently healthy horses at selected farms that represent the geographic distribution of the equine population in Israel. Because *R. equi* prevalence in Israel has never been evaluated, sample size was calculated using the estimation that the Israeli equine population consists of approximately 30 000–40 000 horses, and the prevalence that was found in a similar survey from Turkey was about 10% seropositivity in subclinically infected horses (Tel et al. 2011). With these data, the required sample size for prevalence of 10% ± 5% was 219 horses (Win Pepi 11.43). Horses were sampled on two occasions. In the first survey, 144 horses were sampled on 13 farms during March–May 2011. In the second survey, 293 horses were sampled on 20 farms during November–December 2014. Seven of the farms were sampled on both occasions. Most of the farms sampled were riding schools, some board privately owned horses, eight of the farms bred some of the horses. On each farm, 3–41 horses were sampled (Figure 1). Data of each horse were collected from farm managers and included sex, breed, age, housing, recent health condition and the presence of foals. Blood was collected from the jugular vein into a sterile vacuum tube without an anticoagulant. Sera were obtained from clotted blood samples by centrifugation (3000 × g for 8 min) and stored at −80 °C until use. Positive control serum for a reference standard was obtained from an immunised horse from the Microbiology Department of the Selçuk University, which was confirmed as positive by iELISA and western blot. Negative serum for reference standard was obtained from a foal before ingestion of colostrum.

### Bacterial strain and antigen preparation

In this study, a whole bacterial cell lysate was used as antigen in our in-house ELISA format (Kaba, Kutschke & Gerlach 2001). The virulent *R. equi* strain ATCC 33701 was used to prepare antigen according to the method described previously (Kaba et al. 2001). Briefly, *R. equi* was cultured on brain–heart infusion agar and incubated for 3 days at 37 °C. Bacterial cells were harvested from the surface of agar with 50 mL sterile phosphate-buffered saline (PBS) (PBS; 150 mM NaCl, 2.5 mM KCl, 1.5 mM KH_2_PO4, 9 mM Na_2_HPO_4_.12 H_2_O, pH 7.4) and centrifuged at 7000 rpm for 10 min. After two more washing steps in PBS, the resulting pellet was re-suspended in 12 mL buffer prepared for antigen (0.5 M Tris pH 6.8; 5.2% sodium dodecyl sulfate [SDS]; 8.7% 2-mercaptoethanol). This suspension was boiled for 10 min and centrifuged at 10 000 rpm for 15 min. The supernatant was used as solid ELISA phase antigen and stored at −20 °C for future use.

The strain used for the preparation of the antigen has been previously shown to include VapA protein (Tel et al. 2011); the solid-phase antigen used also included a surface-exposed protein-encoded VapA gene. In this assay, antigen did not comprise solely of VapA protein, since increasing the specificity of the antigen will lead to a decrease in the sensitivity of the assay. This study did not aim to detect the infected animal, but to demonstrate anti-*R. equi* antibody in adult horses. The presence of anti-VapA antibodies has, however, been reported in clinically healthy foals (Takai et al. 1996).

### Enzyme-linked immunosorbent assay for the detection of anti-***Rhodococcus equi*** antibodies

The working dilutions of the horseradish peroxidase-conjugated protein A/G (ImmunoPure, Pierce Lab), antigen preparations and positive and negative sera were determined previously with checkerboard titrations to achieve the highest positive-to-negative ratio with the lowest background reading (1:10 000). The antigen diluted in 0.06 M sodium carbonate buffer (pH 9.6) was coated onto polystyrene plates (Nunc 2-69620, Denmark), 100 µL/well, incubated overnight at 4 °C and then hand washed five times in 0.01 M PBS containing 0.05% Tween 20, pH 7.2 (PBS/Tween). Control and test sera were diluted 1:100 in PBS/T, 100 µL/well, for 1 h at room temperature (RT). After five washes in PBS/T, horseradish peroxidase-conjugated protein A/G was added at 100 µL/well and incubated for 1 h at RT. Finally, after five washes in PBS/T, the colour reaction was developed by adding 100 µL/well of chromogenic solution containing 0.4 mg/mL of o-phenylenediamine dihydrochloride (OPD tablets, Sigma, St. Louis, USA) in 0.05 citrate buffer substrate (pH 5.0) with 0.004% (v/v) H_2_O_2_. The plates were shaken continuously on an orbital shaker in the dark for 15 min. The optical density (OD) measurements were made at 450 nm using a microplate reader (VERSAmax 3.13/B2573). Results were presented in per cent positivity (P%) according to the formula: Mean of duplicate test sample OD-Mean negative control OD/Mean positive control OD-Mean negative control OD × 100. The cut-off value for positive samples was set as the mean OD of the negative control plus 3 standard deviations (s.d.), which corresponded with 20% seropositivity (Tel et al. 2011).

### Statistical analysis

Statistical analysis was performed to detect potential risk factors for the presence of *R. equi* antibodies. Association with nominal independent variables was assessed by using the χ^2^ test, and odds ratios (ORs) were calculated. Association with quantitative parameters was assessed using the *t*-test. Association between variables was considered statistically significant when the *p*-value was less than 0.05. All significant parameters in the univariate analysis were included in a multivariate analysis, using a forward-stepwise model. To control potential confounders, the data were also analysed using generalised estimating equation with a logit link function, with the farm set as a subject (i.e. random variable) and with an exchangeable working correlation matrix. The analysis was performed using SPSS 22.0^®^ and Win Pepi 11.43^®^ statistical software.

## Results

### Study population of the 2011 survey

During March–May 2011, 144 horses were sampled on 13 farms (Figure 1), varying from 3 to 28 horses on each farm (mean = 10.77 ± 6.09, median = 10). Five of these farms (39% of the sampled horses) are breeding farms where foals are born annually. About 51.2% of the horses were from farms in central Israel, 35.4% from the north and 12.5% from the south. Eighty-three (57.6%) of the sampled horses were mares, 57 (39.6%) were geldings and 4 (2.8%) were stallions. Most of the horses (75 horses, 52.1%) were mixed breeds, and the rest were Arabians (*n* = 22; 15.3%), Quarter horses (*n* = 18; 12.5%), ponies (*n* = 13; 9%), Appaloosas (*n* = 10; 6.9%), Warmbloods (*n* = 5; 3.5%) and one Thoroughbred (0.7%). Ages ranged between 3 months and 30 years, with a mean of 10.9 ± 5.7 years. Most of the horses (75.7%) were between 4 and 15 years. Nineteen of the horses (13.2%) were housed in stalls, 83 (57.6%) in paddocks and 42 (29.2%) on pastures.

### Study population of the 2014 survey

During November–December 2014, 293 horses were sampled on 20 farms (Figure 1). Between 6 and 41 horses were sampled on each farm (mean = 14.65 ± 9.67, median = 10.5). Five of these farms (27.6% of the sampled horses) bred some of the mares and have foals annually. Half of the horses (50.5%) were from farms in northern Israel, 17.4% from the centre, 16% from the south and 16% from the Golan Heights. One hundred and thirty-eight horses (47.1%) were mares, 147 (50.2%) were geldings and 7 (2.4%) were stallions. Most of the horses (212 horses, 72.4%) were mixed breeds and the rest were Quarter horses (*n* = 39; 13.3%), Paint horses (*n* = 9; 3.1%), ponies (*n* = 9; 3.1%), Arabians (*n* = 6; 2%), Appaloosas (*n* = 4; 1.4%), Tennessee walking horses (*n* = 4; 1.4%) and one or two horses of each of the following breeds: Missouri fox trotter, Shire, Tinker, Warmblood and Thoroughbred. Horse ages ranged between 9 months and 30 years, with a mean age of 10.5 ± 5.5 years. Most of the horses (73.4%) were aged between 4 and 15 years. Seventy-three horses (24.9%) were housed in stalls, 90 horses (30.7%) in paddocks and 130 (44.4%) on pasture.

### *Rhodococcus equi* seroprevalence and risk factors

Of the 144 horses sampled during spring 2011, 11 (7.6%) were found seropositive with ELISA, all of which originated from one farm. The farm (*P* = 0.001), geographical area (north, *P* < 0.001, all positive horses), housing (pasture, *P* < 0.001, all positive horses), horse breed (Appaloosa, *P* = 0.003, OR = 21.33, 95% confidence interval [CI]:5.1–89.25) and horse colour (appaloosa, *P* = 0.001, OR = 31.25, 95% CI:7.59–128.66) were factors found to be associated with seropositivity in univariable analysis. Multivariable statistical analysis found only the farm (*P* = 0.003) and housing (*P* = 0.013) to be significantly associated with *R. equi* exposure. Because all positive horses originated from a single farm, naturally associations between variables exist. This farm was located in the north of Israel, raises mainly Appaloosas and keeps all horses on pasture (all associations: *P* < 0.001). When using a generalised estimating model to control the farm as a confounder, no significant associations were found.

Of the 293 horses sampled during autumn 2014, 15 (5.1%) were found seropositive for *R. equi* antibodies via ELISA. Seropositive horses were identified at 7/20 (35%) farms. No significant associations with potential risk factors were identified.

### Seroconversion

Forty-nine horses from seven farms were sampled both during 2011 and 2014. Comparing the results at the farm level, the only farm that had positive horses during 2011 also had the highest prevalence (14.6%) in 2014. Of the remaining six farms that were sampled twice and were negative in 2011, seropositive cases were identified on two (33.3% seroconversion at the farm level).

Of the 49 horses that were sampled twice, 42 were seronegative on both occasions, two stayed seropositive on both occasions, four horses that were positive in 2011 were negative in 2014 and one horse (2.3%) seroconverted from negative to positive between 2011 and 2014. The horse that showed seroconversion was sampled at a farm that did not have any seropositive cases in 2011 and was the only seropositive horse (1/15, 6.7%) in 2014.

## Ethical considerations

Blood collections were performed with owners’ consent, and the study was approved by the Internal Ethics Review Committee of the Koret School of Veterinary Medicine, The Hebrew University of Jerusalem.

## Discussion

*Rhodococcus equi* is an important pathogen that can cause severe disease in foals. Furthermore, its zoonotic potential makes it even more important. Early recognition is crucial for successful intervention and treatment. Extended antibiotic therapy is indicated in clinical cases, and increased recognition of the development of macrolide-resistant *R. equi* makes it even harder to treat (Cohen 2014). Better understanding of *R. equi* ecology and epidemiology will increase clinician awareness and is therefore warranted. Definitive diagnosis of the disease is based on the culture of the organism from tracheal wash fluid and considered as the ‘gold standard’. However, it has some disadvantages, including difficulty in sampling and low sensitivity because of possible prior antibiotic administration and the presence of other pathogenic bacteria (Giguère & Prescott 1997). Therefore, serological tests have also been used extensively for diagnosis of the disease and/or to detect specific antibodies to the agent (Giguère et al. 2003; Takai, Kawazu & Tsubaki 1985). Positive serology may indicate exposure to *R. equi*, but not necessarily current infection and in most cases cannot discriminate between infections with virulent or avirulent strains or between clinical or subclinical infection (Cohen 2014; Giguère & Prescott 1997; Giguère et al. 2011b). In this study, we aimed to determine the seroprevalence of anti-*R. equi* antibodies in subclinically infected adult horses in Israel to indirectly indicate the level of exposure of foals, which we had very limited access to because of the low numbers and lack of cooperation from the owners.

The prevalence of *R. equi* exposure in healthy adult horses as seen in this study appears to be low. The observed seroprevalence of 7.6% in 2011 and 5.1% in 2014 did not differ statistically (*P* = 0.29). This is somewhat surprising because we speculated that the local climate, which is warm and dry most of the year, and the management, which is based on many farms on sandy paddocks, would result in higher seroprevalence. On the other hand, the limited number of clinical cases does support the results. Other serological surveys of healthy horses demonstrated slightly higher prevalences (11.7% and 14.8% of horses in different parts of Turkey, 11% in Japan and 13.45% of foals in Italy), although only the results from north-west Turkey (14.8%) (Attili et al. 2006) were statistically different from our findings (*P* = 0.023) (Attili et al. 2006; Cuteri et al. 2003; Sanada et al. 1992; Tel et al. 2011).

Although the population at risk for *R. equi* clinical infection is mainly young foals, this survey consisted almost entirely of adult horses, with very few horses under 1 year of age. The equine breeding industry in Israel is small, large-scale breeding facilities are scarce and owners are reluctant to allow sampling of foals that are not regularly handled. Most of the previous studies included some (Attili et al. 2006; Tel et al. 2011) or only (Cuteri et al. 2003) foals, although seroprevalence did not appear to be influenced by age (Attili et al. 2006; Tel et al. 2011). Serological tests used for the detection of *R. equi-*specific antibodies were designed to evaluate recent exposure. Because the level of antibodies in individual horses was not found to reflect the epidemiological status of the farm (Witkowski et al. 2012), ELISA results in this study and similar surveys were treated as a dichotomous parameter, to indicate exposure (Attili et al. 2006; Sanada et al. 1992; Tel et al. 2011). Little is known about the kinetics of antibody concentrations over time, but the fact that seroprevalence did not correlate with age (Attili et al. 2006; Tel et al. 2011) implies that adult horses may serve as sentinels for exposure in foals. Similar seroprevalences at both time points support the assumption that this bacterium is endemic in Israel, with probable endemic stability. This is also supported by the small number of horses that seroconverted after 3 years. The fact that some farms that were negative in 2011 had positive horses in 2014 may be ascribed to the fact that not all horses were sampled on both occasions (because of owner consent) or of new horses that were introduced to these farms during that time period.

The factors that influence *R. equi* incidence and acquisition of virulence are not fully understood. Exposure of foals to the bacterium early in life in combination with their immunological status seems to be crucial (Cohen 2014; Reuss & Cohen 2015). The amount of bacteria in soil and faeces does not appear to be associated with clinically infected foals, whereas factors that influence the amount of airborne particles (animal density and housing in stalls) do seem to correlate with clinical disease (Cohen 2014; Muscatello 2012a; Reuss & Cohen 2015). Our findings did not show an association between stable management and seroprevalence, and the only positive farm included at both time points keeps the horses on pasture. In addition, the presence of VapA protein is necessary, but not sufficient to cause disease in foals (Reuss & Cohen 2015). The detection of antibodies against a virulent strain may reflect the potential for acquisition of such a strain by susceptible foals, but does not necessarily correlate with its virulence *de facto* and the chance to develop clinical disease.

The use of a serological survey of adult horses to represent the risk of foals to acquire *R. equi* has several limitations. As mentioned before, seropositive results do not indicate actual infection, but only exposure to the bacterium. Also, because crude antigen was used in the ELISA, although prepared from a virulent strain, positive results may not necessarily indicate exposure to a virulent strain. Adult horses are usually not susceptible to clinical infection, and exposure in this population does not directly correlate with disease. The use of foal serum prior to nursing as a negative control also has some limitations because it lacks antibodies, while sera of negative adult horses have a nonspecific antibodies that may influence the reading. Nevertheless, because no documentation of *R. equi* exposure in Israel exists up to date, the results of this survey may provide some knowledge about the epidemiology of this bacterium.

Identification of *R. equi-*positive farms is important to raise the awareness of attending clinicians to early diagnosis and treatment of clinically affected animals. Because no effective prevention or control methods exist, the focus on farms where clinical cases occurred should be on early diagnosis using routine screening of foals by haematology, imaging or both. Although these methods also lack in specificity and may lead to unnecessary treatment in some cases, there is no preferable alternative to date (Cohen 2014).

## Conclusion

The prevalence of *R. equi* exposure observed in healthy adult horses did not differ statistically between 2011 and 2014 and was found to be 7.6% and 5.1%, respectively. Although low, the detected seroprevalences indicate that the causative organism continues to be present on Israeli farms and poses a potential risk to susceptible foals. Clinicians should be aware of the presence of this pathogen on farms for early detection and treatment of clinically infected foals and immunocompromised people.
